# Erythema Nodosum as the Primary Manifestation of Acute Histoplasmosis in a Pediatric Patient From an Endemic Region

**DOI:** 10.7759/cureus.92658

**Published:** 2025-09-18

**Authors:** Emily Saurborn, William T Snider, Hayley Weese, Joseph Evans, Shane Cook, Jacob T Kilgore

**Affiliations:** 1 Dermatology, Marshall University Joan C. Edwards School of Medicine, Huntington, USA; 2 Pediatrics, Marshall University Joan C. Edwards School of Medicine, Huntington, USA; 3 Pediatric Infectious Diseases, Marshall University Joan C. Edwards School of Medicine, Huntington, USA

**Keywords:** erythema nodosum, fungal infection, histoplasmosis, pediatric dermatology, pediatric infectious disease

## Abstract

Erythema nodosum (EN) is a common form of septal panniculitis that typically presents with painful, erythematous nodules on the lower extremities. While most cases are idiopathic, EN can be triggered by infection, medications, autoimmune and inflammatory pathologies, recent immunization, or malignancy. Commonly, EN has been associated with fungal infections, including *Histoplasma capsulatum*, a dimorphic fungus endemic to the Ohio and Mississippi River Valleys. Although pulmonary involvement is the most common manifestation of the fungal infection, extrapulmonary presentations can occur. We present a case of a nine-year-old immunocompetent male patient from an endemic region who developed EN in the absence of any pulmonary symptoms or radiographic abnormalities. Serologic testing was notable for elevated *H. capsulatum *antibodies, and the patient reported complete resolution with supportive care alone. This case highlights the importance of considering fungal etiologies while maintaining a broad differential diagnosis when evaluating EN, especially in endemic areas.

## Introduction

Erythema nodosum (EN), a common type of septal panniculitis, typically presents with painful, erythematous nodules located on the pretibial areas [1,2]. While 50% of all cases of EN are idiopathic, known triggers may include infections, medications, pregnancy, recent vaccination, autoimmune conditions, and malignancy [2]. Commonly associated bacterial infections include group A streptococcal (GAS) and *Bartonella* [1]. Among fungal pathogens, endemic fungi such as *Coccidioides immitis* (*C. immitis*), *Histoplasma capsulatum *(*H. capsulatum*), and *Blastomyces dermatitidis *(*B. dermatitidis*) are known triggers [1]. Diagnosis is clinical, and most cases of EN are self-limited, requiring only supportive care.

*Histoplasma capsulatum* is a dimorphic fungus found in bird and bat feces-contaminated soil, notably in the Ohio and Mississippi River Valleys [3-5]. Inhalation of fungal spores typically leads to pulmonary infection, ranging from mild pneumonitis to acute respiratory distress syndrome [6,7]. Primary infections in immunocompetent patients are usually asymptomatic. When symptomatic, non-pleuritic chest pain, high fevers, headache, and nonproductive cough are reported as the prominent symptoms [3]. Extrapulmonary manifestations, including EN, arthralgias, and erythema multiforme, are seen in nearly 6% of immunocompetent individuals [3]. Chest imaging typically reveals patchy diffuse pneumonitis with hilar lymphadenopathy [3]. In immunocompromised individuals, infection can become disseminated, leading to severe disease, multisystem organ involvement, and sepsis [6].

While EN has been associated with histoplasmosis, it atypically presents as the primary manifestation in pediatric patients. This case highlights the uncommon presentation of histoplasmosis presenting exclusively with EN alongside fever, arthralgias, and weight loss in the absence of any pulmonary findings.

## Case presentation

**Figure 1:**
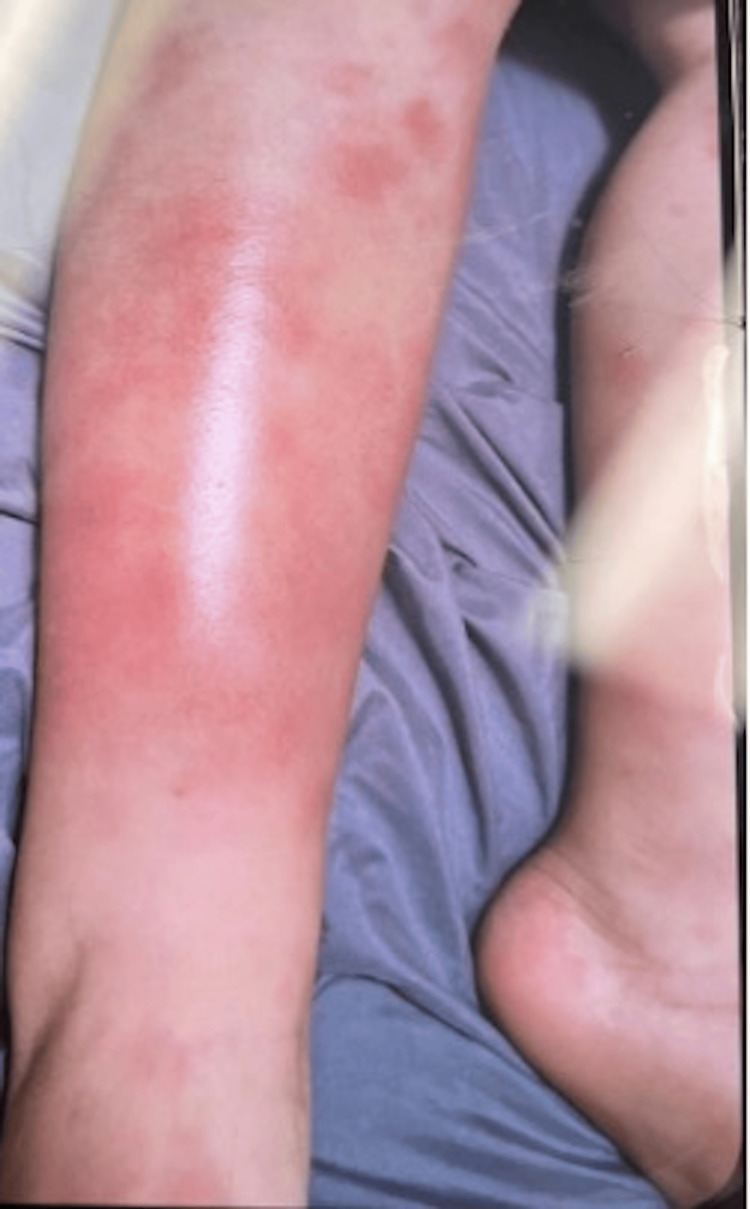
Nodules present with expanding blanchable erythema and ill-defined borders

A nine-year-old male patient presented to his pediatrician for new-onset painful, erythematous, raised nodules on his legs and left arm. Symptoms began after staying the night at a friend’s house, in which the family reported potential bed bugs in the home, raising initial concern for exposure. Upon further questioning, he reported increased fatigue and headaches but denied any fever. Two days later, the patient reported worsening nodules that now itched and were painful. He remained afebrile and continued to deny any cough, congestion, or rhinorrhea. No visual changes, neck stiffness, or altered mental status were reported. Physical examination revealed warm, tender, nodular lesions with blanchable erythema and ill-defined borders (Figure 1). The patient was advised to start nonsteroidal anti-inflammatory drugs (NSAIDs) and follow up in two weeks.

**Figure 2:**
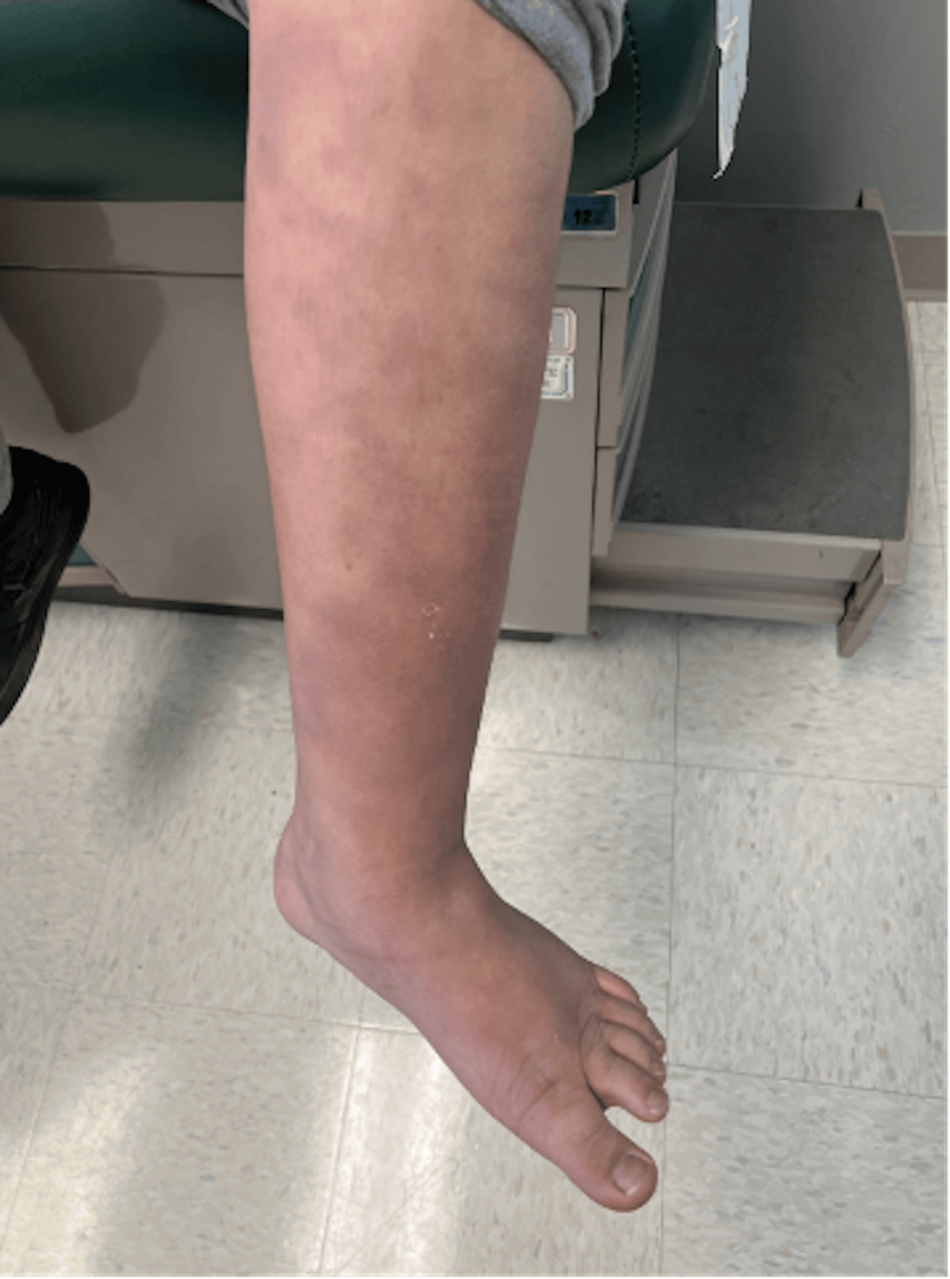
Violaceous livedo racemosa-like rash present on the bilateral lower extremities

During the two-week follow-up, the rash had evolved into a violaceous livedo racemosa-like pattern that now involved the thighs, buttocks, and lower back in addition to the extremities (Figure 2). The patient denied any mucosal involvement but did report pruritus and pain, most prominent at night. On review of systems, he endorsed lower back and ankle pain, temperature maximum at night (100°F), and a four-pound weight loss. He also noted a recurring painful lesion in his right axilla that occasionally drained and produced a foul odor. He continued to deny any cough, congestion, or respiratory symptoms.

**Figure 3:**
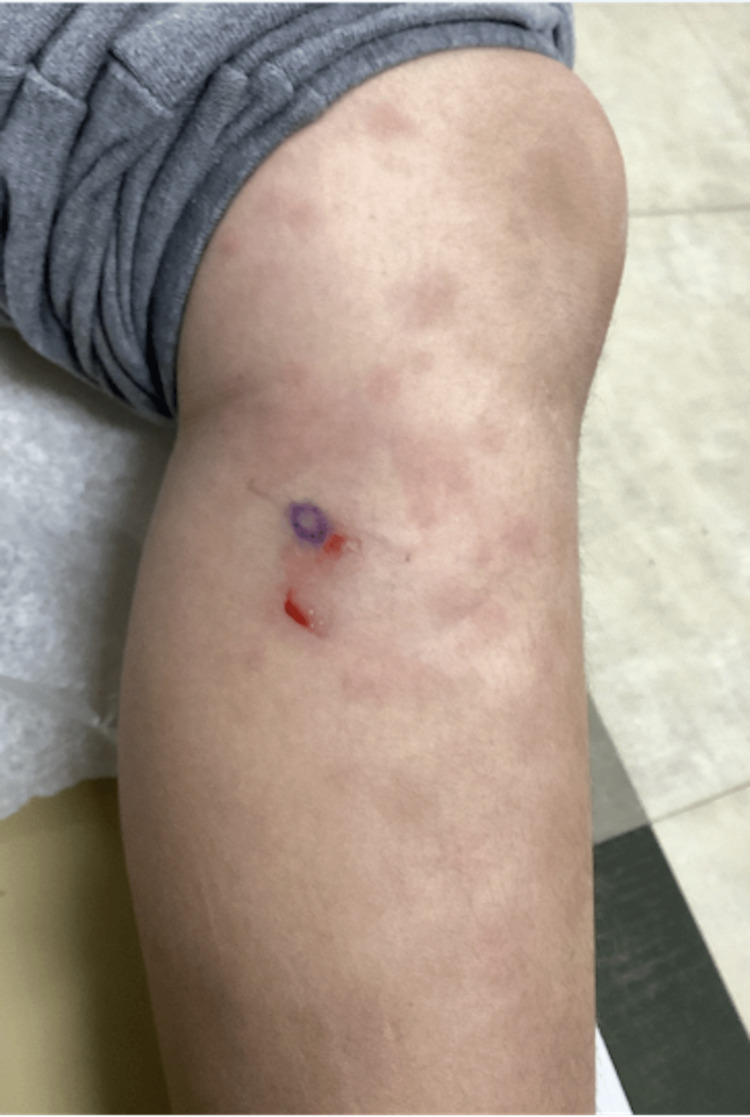
Diffuse erythematous nodules present on the lower extremity The image depicts the punch biopsy site.

The patient was referred to dermatology, and at this time, differential diagnosis included EN, possibly secondary to hidradenitis suppurativa (HS), polyarteritis nodosa, macular lymphocytic arteritis (MLA), or vasculitis. Punch biopsy (Figure 3) revealed septal panniculitis consistent with EN. The patient was also diagnosed with HS and started on a topical wash. Additionally, the patient was referred to rheumatology, and further laboratory results were significant for an elevated C-reactive protein (CRP) of 11.2 (normal: <10 mg/L), erythrocyte sedimentation rate (ESR) of 64 mm/hour (normal: 0-20 mm/hour), and complement C3 level of 213 mg/dL (normal: 90-180 mg/dL). Infectious and autoimmune workup was initiated, and serologic testing, including both complement fixation and immunodiffusion, revealed elevated *H. capsulatum* antibody levels (Table 1). The chest radiograph was negative for any signs of acute infection.

**Table 1:** Results from autoimmune and infectious workup completed by rheumatology RF: rheumatoid factor, CCP: cyclic citrullinated peptide, ENA panel: extractable nuclear antigen panel, DsDNA: double-stranded DNA, TB: tuberculosis, HLA-B27: human leukocyte antigen B27

Laboratory finding	Result	Reference range
RF, CCP	Negative	*≤*14 IU/mL
ENA panel	Negative	*≤*10 IU/mL
DsDNA	Negative	*≤*10-30 IU/mL
TB	Negative	-
HLA-B27	Negative	-
*Bartonella henselae*/*Bartonella quintana* serum serologies	Negative	IgG or IgM titer: <1:64 or <1:128
*Histoplasma capsulatum* serum serologies	Positive, complement fixation 1:256/H&M bands present	-

The patient was evaluated by the pediatric infectious diseases team. As the patient was showing signs of improvement, it was decided to continue NSAIDs without the addition of antifungal therapy. Upon follow-up, he reported complete resolution of cutaneous and systemic involvement and continued to deny any pulmonary symptoms.

## Discussion

This case illustrates an uncommon presentation of acute histoplasmosis manifesting as EN and mild constitutional symptoms in a pediatric patient. Pulmonary involvement is expected; however, extrapulmonary signs, including EN, may occur secondarily as a hypersensitivity reaction to the fungus rather than fungal invasion into the tissues. The pathophysiology of EN involves a cell-mediated immune response leading to pro-inflammatory cell infiltration within the subcutaneous septa. Although not specific to histoplasmosis, EN has been reported as a concurrent finding, as demonstrated during the 1978-1979 Indianapolis outbreak, in which 4.1% of individuals presented with EN [8].

Symptomatic pediatric histoplasmosis most commonly presents with pulmonary involvement, and there is scarce literature on the primary presentation of EN in the absence of other pulmonary findings. In 1960, the first case of EN secondary to histoplasmosis was reported by Little and Steigman [9]. A two-year-old female patient presented for a three-day history of painful pretibial nodules after recent exposure to tuberculosis (TB). Skin testing was negative for TB; however, serum antibody levels of *H. capsulatum* were elevated. Chest imaging was negative for any signs of acute infection. Little and Steigman described three additional cases of EN as a primary manifestation of histoplasmosis; however, upon further workup, pulmonary findings were evident on imaging [9]. Further, in 1966, Medeiros et al. reported that nearly half of all patients with histoplasmosis and EN lacked pulmonary findings on chest imaging [10].

The differential diagnosis in this case was broad, including many infectious and inflammatory conditions such as hidradenitis suppurativa (HS), given his recent diagnosis. Although EN is not commonly associated with HS, it may occur as part of the broader spectrum of inflammatory dermatosis, notably in the setting of systemic immune activation [11]. In this case, however, the diagnosis of HS was a coincidental finding. Other considerations included autoimmune vasculitis, given his family history of alopecia and rheumatoid arthritis, and infectious causes such as* Bartonella* due to recent exposure to a new kitten and blastomycosis due to endemicity and skin involvement. These were deemed less likely based on available testing results. Treatment for EN in this scenario is often supportive and includes NSAIDs, bed rest, and leg elevation [2,7].

## Conclusions

This case highlights an uncommon presentation of histoplasmosis presenting solely as EN in an immunocompetent pediatric patient without evidence of pulmonary involvement. It is important for clinicians to consider fungal infections as a source of EN in endemic areas. Typically, conservative management is sufficient in immunocompetent patients with histoplasmosis-associated EN, and overtreatment should be avoided. Early involvement of a multidisciplinary team can ensure a timely diagnosis and appropriate management in atypical cases.

## References

[1] Hafsi W, Badri T (2022). Erythema nodosum. StatPearls [Internet].

[2] Leung AK, Leong KF, Lam JM (2018). Erythema nodosum. World J Pediatr.

[3] Akram SM, Koirala J (2023). Histoplasmosis. StatPearls [Internet].

[4] Mukherjee T, Basu A (2015). Disseminated histoplasmosis presenting as a case of erythema nodosum and hemophagocytic lymphohistiocytosis. Med J Armed Forces India.

[5] Wheat LJ, Freifeld AG, Kleiman MB, Baddley JW, McKinsey DS, Loyd JE, Kauffman CA (2007). Clinical practice guidelines for the management of patients with histoplasmosis: 2007 update by the Infectious Diseases Society of America. Clin Infect Dis.

[6] Kauffman CA (2007). Histoplasmosis: a clinical and laboratory update. Clin Microbiol Rev.

[7] Pappas P, Lentz RJ, Stover KR (2025). 2025 clinical practice guideline update by the Infectious Diseases Society of America on histoplasmosis: treatment of mild or moderate acute pulmonary histoplasmosis in adults, children, and pregnant people. Clin Infect Dis.

[8] Wheat LJ (1992). Histoplasmosis in Indianapolis. Clin Infect Dis.

[9] Little JA, Steigman AJ (1960). Erythema nodosum in primary histoplasmosis. J Am Med Assoc.

[10] Medeiros AA, Marty SD, Tosh FE, Chin TD (1966). Erythema nodosum and erythema multiforme as clinical manifestations of histoplasmosis in a community outbreak. N Engl J Med.

[11] Scheinfeld N (2013). Diseases associated with hidranitis suppurativa: part 2 of a series on hidradenitis. Dermatol Online J.

